# The induction and consequences of Influenza A virus-induced cell death

**DOI:** 10.1038/s41419-018-1035-6

**Published:** 2018-09-25

**Authors:** Georgia K. Atkin-Smith, Mubing Duan, Weisan Chen, Ivan K. H. Poon

**Affiliations:** Department of Biochemistry and Genetics, La Trobe Institute for Molecular Science, La Trobe University, Melbourne, VIC 3086 Australia

## Abstract

Infection with Influenza A virus (IAV) causes significant cell death within the upper and lower respiratory tract and lung parenchyma. In severe infections, high levels of cell death can exacerbate inflammation and comprise the integrity of the epithelial cell barrier leading to respiratory failure. IAV infection of airway and alveolar epithelial cells promotes immune cell infiltration into the lung and therefore, immune cell types such as macrophages, monocytes and neutrophils are readily exposed to IAV and infection-induced death. Although the induction of cell death through apoptosis and necrosis following IAV infection is a well-known phenomenon, the molecular determinants responsible for inducing cell death is not fully understood. Here, we review the current understanding of IAV-induced cell death and critically evaluate the consequences of cell death in aiding either the restoration of lung homoeostasis or the progression of IAV-induced lung pathologies.

## Facts

- The mechanism and consequence of IAV-induced cell death are still debatable.
- IAV can induce cell death through apoptosis, necrosis, necroptosis and possibly pyroptosis.
- The mechanism and outcome of IAV-induced cell death are likely to be cell type and/or viral strain dependent.
- IAV-induced apoptosis is likely to play a pro-viral role and aid IAV pathogenesis.
- The generation of dead cells and their debris during IAV infection may contribute to antigen presentation and timely removal is essential to aid disease resolution.

## Open Questions

- Which factors ultimately determine the pathway of IAV-induced cell death?
- Do apoptotic and necrotic debris have different roles during IAV infection?
- Could targeting cell death during IAV infection be an effective anti-viral therapeutic?

## Introduction

Apoptosis is a key form of programmed cell death, characterised by two distinct pathways including the cell intrinsic and extrinsic pathways^1^. The intrinsic or mitochondrial-dependent pathway involves the activation of the pro-apoptotic molecules Bax and Bak, which are able to induce permeabilisation of the outer mitochondria membrane^2^. This permeabilisation allows the release of cytochrome c, formation of the apoptosome and activates the executor caspases which dismantle the cell^3^. The extrinsic pathway is induced by ligands which bind to death receptors including Fas located on the plasma membrane, and results in caspase 8 activation^4^. Apoptosis is characterised by hallmarks such as DNA fragmentation, cell surface phosphatidylserine (PtdSer) exposure, plasma membrane blebbing and apoptotic body formation^5^. As the plasma membrane remains intact during apoptosis, apoptotic cell death is generally considered as an anti-inflammatory process. However, the persistence of uncleared apoptotic cells can result in rupture of the plasma membrane and the release of proinflammatory intracellular contents through secondary necrosis^6,7^. Although membrane permeabilisation during secondary necrosis has previously been thought to be an unregulated process, recent studies suggest that an N-terminal fragment generated from caspase-cleaved gasdermin E/DFNA5 may actively mediate this process^8,9^. In contrast, primary necrosis is directly induced by exposure to an array of stimuli such as antimicrobial peptides^10^, bacterial endotoxin^11^ and heat shock^12^. Finally, similar to necrosis, necroptosis is an inflammatory form of cell death characterised by the formation of large necrotic blebs and membrane permeabilisation^13^. However, necroptosis is a highly controlled process regulated by a series of proteins including RIPK1/3 and MLKL, for a detailed review see Pasparakis et al.^14^.

**Fig. 1:**
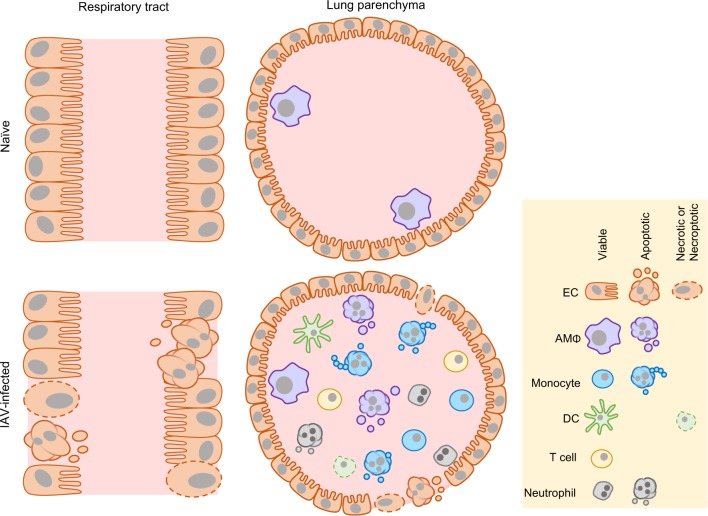
IAV-induced cell death. During homoeostatic conditions, airway and alveolar epithelial cells (ECs) line the surfaces of the lung and AMΦs can be found within the lung parenchyma. IAV infection induces apoptosis and necrosis of ECs, compromising the integrity of the epithelial cell barrier. Furthermore, immune cells including monocytes, DCs and neutrophils infiltrate into the lung parenchyma and in turn are susceptible to IAV-induced apoptosis and necrotic-like cell death

One of the many factors that can modulate the cell death process is viral infection, in particular Influenza A virus (IAV). Influenza infection significantly impacts health worldwide with the World Health Organisation estimating ~250,000–500,000 infection-related deaths in 2016. IAV belongs to one of three influenza genera (including A, B and C) of the *Orthomyxoviridae* family and is a segmented negative-sense RNA virus. The 8 gene segments of IAV encode for 13 known proteins (Table 1) which are able to undergo rapid mutation^15,16^. IAV infection induces rapid immune cell infiltration into the lung parenchyma and thus, an array of cell types are exposed to IAV and susceptible to infection-induced death including apoptosis^17^, primary necrosis^18^ and necroptosis^19^ (Fig. 1). The best-described mechanism of IAV-induced cell death is apoptosis, which has been observed in many cell types including monocytes^17^, macrophages^20^ and epithelial cells^21^ under both in vitro and in vivo conditions. Here, we review the current understanding of IAV-induced cell death and discuss how cell death impacts disease resolution and IAV pathogenesis.

**Table 1:** Role of IAV proteins in IAV pathogenesis and host cell death

IAV Protein	Primary viral function	Role in cell death
**NP**	–Nucleocapsid protein which provides virion structure –Mediates genome replication through RNA binding activity	–Possibly inhibits anti-apoptotic host proteins such as AP15^35^
**NS1**	–Antagonises host IFN response –Mediates vRNA synthesis, mRNA splicing and translation	–Prevents the early induction of apoptosis by inhibiting pro-apoptotic proteins such as Scribble^26,28^ –Induces apoptosis downstream of FasR^21^
**NS2 (NEP)**	–Mediates export of viral RNA from the nucleus to the cytoplasm	–
**PA**	–Part of the RNA polymerase complex, required for RNA synthesis	–
**PB1**	–Part of the RNA polymerase complex, required for RNA synthesis	–
**PB2**	–Part of the RNA polymerase complex, required for RNA synthesis	–
**PA-X**	–Impairs cellular host gene expression	–
**PB1-F2**	–Intrinsically induces apoptosis	–Mediates permeabilisation of the mitochondrial membrane through ANT3 and VDAC1^29^
**PB1-N40**	–Currently unclear	–
**NA**	–Cleaves sialic acid to release viral progeny	–
**HA**	–Mediates host cell entry by binding membrane receptors	–Some variants may impair IAV-induced necroptosis^19^
**M1**	–Provides structure and stability to the virion	–
**M2**	–Ion channel which aids viral assembly and budding	–Interacts with autophagy regulators and blocks autophagosome fusion, in turn enhancing apoptosis^41^

### Apoptosis inhibition by IAV

It is well documented that IAV can modulate cell death pathways however, the specific molecular mechanisms by which IAV regulates apoptosis is complex and yet to be fully defined. As viral replication must be completed before dismantling of the cell through apoptosis, the expression of anti-apoptotic viral proteins may facilitate viral propagation prior to cell death. In line with this, the multifunctional IAV protein NS1 has been implicated in suppressing the host interferon response^22,23^, and both promoting^21^ (as discussed below) and inhibiting^24,25^ apoptosis (Table 1). Initial studies found that deletion of NS1 resulted in the rapid and efficient induction of apoptosis in IAV-infected kidney epithelial cells in vitro^25^. As the anti-apoptotic properties of NS1 were absent in cells lacking IFN-α/IFN-β, NS1 may inhibit host cell apoptosis through a type I IFN-dependent mechanism^25^. Although the precise mechanism of how NS1 can limit apoptosis is not fully understood, NS1 may directly interact with and inhibit pro-apoptotic host factors through a N-terminal PDZ-binding motif^26^. For example, NS1 can bind the pro-apoptotic protein Scribble through its PDZ-domain and thus limit host cell apoptosis^24^. It is interesting to note that the PDZ-binding motif of NS1 is also required for efficient viral propagation, as mutation of this domain can significantly reduce viral titres^24^. Taken together, NS1 may prevent the early induction of apoptosis, consequentially aiding IAV pathogenesis.

### Intrinsic-induction of apoptosis by IAV

**Fig. 2:**
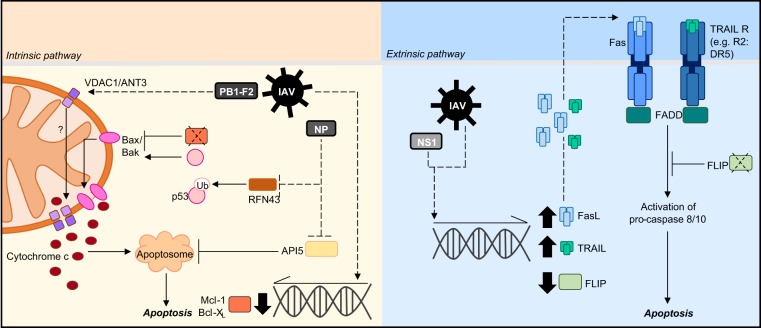
Molecular mechanisms of IAV-induced apoptosis. IAV can induce host cell apoptosis through either the intrinsic or extrinsic pathway. To induce apoptosis via the intrinsic pathway, IAV infection can promote the downregulation of anti-apoptotic proteins (Mcl-1/Bcl-X_L_) and p53 stabilisation. In turn, these mechanisms activate bax/bak to facilitate mitochondrial membrane pore formation and allow cytochrome c release. The IAV protein PB1-F2 can interact with VDAC1 and ANT3 to promote mitochondria outer and inner membrane permeabilisation and the release of pro-apoptotic molecules. Additionally, IAV infection can inhibit the anti-apoptotic protein API5 which usually functions to restrict apoptosome formation. IAV infection can also induce apoptosis through the extrinsic pathway by increasing the expression of death receptor ligands including FasL and TRAIL. Furthermore, infection can downregulate the expression of the anti-apoptotic protein FLIP which usually functions to limit Fas-mediated apoptosis. Dotted lines refer to IAV-targeted processes, solid lines refer to host cell processes

Although IAV can induce both intrinsic and extrinsic apoptosis, the best-described mechanism comes from the discovery of the H1N1-IAV protein PB1-F2 which is produced through an alternative reading frame of the IAV genome (Fig. 2)^17^. PB1-F2 can localise to the mitochondria and interact with various mitochondrial membrane proteins including ANT3 (inner membrane) and VDAC1 (outer membrane) to facilitate mitochondrial membrane permeabilisation and cytochrome c release^17,27^. Interestingly, although the loss of PB1-F2 had no effect on viral replication^17^, IAV-PB1-F2^−/−^ virions were cleared more efficiently and lessened disease severity in mice^28^. Although this highlights the importance of PB1-F2-induced apoptosis in aiding viral pathogenesis, the pro-apoptotic role of PB1-F2 is viral strain and cell-type dependent. PB1-F2 activity and mitochondrial co-localisation, as well as overall viral pathogenicity differs between viral strains which contain PB1-F2 variants/truncations, such as H1N1, H3N2 and H5N1^29^.

Furthermore, IAV-induced apoptosis is likely to be dependent on Bax/Bak activity^30^, and/or through downregulating anti-apoptotic factors such as Mcl-1 and Bcl-X_L_^31^. Additionally, the IAV protein NP may also exhibit pro-apoptotic roles as expression of NP in the epithelial cell line A549 is sufficient to intrinsically induce apoptosis^32^. Although the mechanism of NP-induced apoptosis is not completely understood, NP can directly interact with the anti-apoptotic host factor API5, preventing downregulation of APAF1 and promote apoptosis^33^. NP may also exert its pro-apoptotic function through the inhibition of the E3 ubiquitin ligase RNF43^34^. As RNF43 can mark p53 for degradation through ubiquitination, the interaction of NP with RNF43 can result in p53 stabilisation and consequently promote apoptosis through Bax/Bak activation^34^.

### Extrinsic-induction of apoptosis by IAV

In addition to the mitochondrial-dependent pathway, IAV can also extrinsically induce apoptosis (Fig. 2). IAV infection can result in the expression of death receptor ligands including FasL and TRAIL^35–37^. The upregulation of FasL expression by IAV (e.g. H1N1) in the epithelial cell line A549 stimulates Fas, activates the apoptotic FADD complex and extrinsically induces cell death^35^. Moreover, IAV infection can also downregulate the expression of the anti-apoptotic factor FLIP, which usually functions to inhibit Fas-mediated apoptosis^35^. Interestingly, enhancing FasL expression significantly increases viral RNA replication, and replication is impaired by the addition of a caspase 8 inhibitor, further supporting the pro-viral role of apoptosis during IAV infection^35^.

In contrast to the anti-apoptotic properties of NS1, expression of IAV (H5N1)-NS1 and not NS2 was also shown to induce characteristic apoptotic DNA fragmentation^38^. Moreover, NS1 expression alone could promote other apoptotic hallmarks including PtdSer exposure and caspase 3 activation^21^. As NS1-induced apoptosis is significantly enhanced in FasL-treated cells, data suggests that NS1 may be functioning downstream of Fas-mediated apoptosis^21^. Although the specific mechanism underpinning NS1-induced apoptosis is unclear, NS1 requires a functional RNA-binding domain to facilitate extrinsically-induced apoptotic cell death^38^.

### Manipulation of autophagy by IAV to promote apoptosis

A variety of IAV strains (H3N2 and H1N1) have also been implicated in manipulating autophagy^39^. Autophagy is characterised by the formation of autophagosomes which capture and degrade cellular components through fusion with the lysosome^40^. However, IAV infected-epithelial cells can contain an accumulation of autophagosomes which do not proceed to form phagolysosomes^39,41^. Moreover, the IAV protein M2 can directly interact with the autophagy regulators Atg5/Beclin-1 in vitro to block autophagosome fusion, preventing the degradation of substrates within the lysosome (Table 1)^39^. Although yet to be fully understood, NS1 has also been implicated in regulating M2-dependent manipulation of autophagy, providing an effective mechanism to evade autophagy-mediated viral clearance^42^. Interestingly, M2-dependent inhibition of autophagy can significantly enhance apoptosis whereby Atg5 or M2 deficiency increases IAV-infected cell viability^39^. Together, this highlights a unique pathway whereby IAV may interfere with autophagy in turn promoting apoptosis, and further supports the pro-viral role of apoptosis during the pathogenesis of IAV^39^.

## IAV infection and necrotic-phenotypes of cell death

In addition to apoptosis, IAV-induced necrosis is also well described in severe human and avian infections. For example, IAV infection (H9N2) of chicken epithelial cells results in necrotic cell death, demonstrated by membrane permeabilisation and the lack of apoptotic characteristics^18^. Similarly, IAV-induced necrosis can be observed in lethal IAV outbreaks including the 2006 IAV (H5N1)-infection which resulted in a mass mortality of birds that exhibited severe necrosis pathology^43^. In addition to avian infections, epithelial cells are commonly known to undergo IAV-mediated necrosis during human infections, likely contributing to the characteristic respiratory tract damage observed in severe infections^44,45^. However, as uncleared apoptotic cells can proceed to secondary necrosis, interpretation of necrotic data should be evaluated carefully as late apoptotic cells may exhibit necrotic characteristics. Furthermore, necroptotic cells also exhibit morphologies indistinguishable from necrotic cells^46^. Therefore, unless the activation of apoptotic and/or necroptotic regulators are evaluated, the induction of cell death solely through primary necrosis cannot be concluded.

Recent findings have now shed light on IAV-induced necroptosis. As mentioned above, necroptosis is mediated by a series of molecular regulators including RIPK1/3 and MLKL^14^. Furthermore, induction of necroptotic cell death is also limited by caspase activation as caspase 8 can cleave and inactivate RIPK1/3^14,47,48^. Therefore, if caspase activity is blocked by a pan-caspase inhibitor, IAV infection of dendritic cells (DCs) can induce a necrotic-like cell death dependent on RIPK3 activity, thus demonstrating the ability of IAV to promote necroptosis^19,49^. Similar to IAV-induced apoptosis, induction of necroptosis by IAV may be viral strain-specific as DCs infected with seasonal IAV (such as A/New Caledonia/20/99) can undergo RIPK3-mediated necroptosis, whereas pandemic IAV (such as A/California/7/2009) can suppress cell death. Although the underlying mechanism is not fully understood, inhibition of necroptosis by pandemic IAV was mediated by the IAV protein HA which exhibits a slight variation between the two IAV strains^19^. Interestingly, as DCs infected with seasonal IAV can undergo necroptosis and release proinflammatory molecules, seasonal infections may be more efficient in inducing an immune response and aiding viral clearance^19^. In line with this, the loss of RIPK3 and subsequent prevention of IAV-induced necroptosis enhanced disease severity and mice mortality during IAV (H1N1)-infection, indicating an anti-viral role of necroptosis^50^. It is important to note that the dsDNA sensor Zbp1 was recently shown to mediate IAV detection and promote RIPK3-mediated necroptosis^51^. However, in contrast to the aforementioned findings, the loss of Zbp1 limits IAV-induced cell death and decreases IAV-induced mortality during IAV (H1N1)-infection. As Zpb1 was also suggested to aid the induction of apoptosis which is likely to play a pro-viral role during IAV infection, it is possible that the reduction in disease severity was a consequence of impaired apoptosis rather than solely necroptosis^51^.

The anti-apoptotic protein cIAP2 may also play a key role in regulating IAV-induced cell death. Although cIAP2 normally functions as a protein ubiquitin ligase which can ubiquitinate caspase 3/7 and inhibit apoptosis, cIAP2 may play alternate roles during IAV infection^52,53^. The absence of cIAP2 during IAV infections in mice results in severe epithelial necroptosis, tissue haemorrhaging and overall enhances IAV lethality^54^. As cIAP2 can also ubiquitinate RIPK1/3 for degradation, during IAV infection cIAP2 deficiency leads to the assembly of the RIPK/FADD necroptosome and thus necroptosis^54–56^. Furthermore, the absence of cIAP2 during IAV infection promotes the expression of the short isoform of FLIP (FLIP_s_), which can inhibit apoptosis and consequentially promote necroptosis^54^. Although an equivalent proportion of epithelial cell death occurred in cIAP2^−/−^ and wild-type IAV-infected mice, the percentage of dead cells containing active caspase 3 was significantly reduced in the cIAP2 deficient mice, indicating that lack of cIAP2 could sway the pathway of cell death to promote non-apoptotic cell death^54^. Taken together, cIAP2 plays a role in the host response during IAV infection to prevent the induction of proinflammatory cell death.

Finally, pyroptosis may also be implicated in IAV infection. Pyroptosis is characterised by the formation of the inflammasome which leads to caspase 1 activation, proinflammatory cytokine processing and release, and membrane permeabilisation through caspase-activated gasdermin D pores (also mediated by caspase 11 activation through the noncanonical pathway)^57,58^. Inflammasome activation is suggested to play a key role in aiding disease resolution during IAV (H1N1) infection, as the lack of inflammasome regulators such as NLRP3 or caspase 1 during mice-infections models can significantly enhance disease lethality^59,60^. The activation of the inflammasome during IAV infection is suggested to be a consequence of viral RNA sensing and/or M2 activity^59–61^. Interestingly, NS1 may directly interact with NLRP3 to suppress the inflammasome activation and the release of IL-1β by human monocyte-derived macrophages^62^. As IL-1β release by caspase 1-activated gasdermin D is usually indicative pyroptosis, it is likely that IAV infection can induce pyroptotic cell death and despite being proinflammatory, may aid disease resolution^51,59,60,63^. However, more accurate conclusions can be drawn from monitoring the induction of cell death and disease severity in mice lacking gasdermin D during IAV infections.

Altogether, IAV is likely to induce a variety of cell death pathways through both direct infection and infection of neighbouring cells. This is clearly evident in vivo as both infected and uninfected cells undergo cell death, highlighting the role of extrinsic factors in mediating cell death during infection (as discussed further below)^54^. As IAV infection can result in multiple cell death outcomes, the overall impact on viral immunity is difficult to conclude. In some circumstances the induction of apoptosis may facilitate viral replication and pathogenesis, whereas necroptosis may favour the host anti-viral response. Such diversity highlights the importance of evaluating key cell death parameters (such as caspases activation, PtdSer exposure, membrane permeability, apoptotic body formation, DNA fragmentation and RIPK3 activation) and considering the time post infection when determining the initiation of a specific cell death pathway. One outstanding question that remains to be determined is the interplay and regulation between pro and anti-cell death IAV proteins. For example, how it is that NS1 could prevent the early induction of apoptosis but allow PB1-F2 to induce cell death at later stages? What is the mechanism that controls such kinetics? Nevertheless, the complexity of IAV-induced cell death may provide an evolutionary-developed mechanism to evade host defence mechanisms.

## Cell type-specific consequences of cell death

Both non-immune and immune cell types are known to undergo IAV-induced cell death via the pathways described above, which in turn may further drive pro-viral or anti-viral responses (Fig. 2). Overall, the beneficial vs. pathological consequences of IAV-induced cell death varies between different cell types.

### Epithelial cell death during IAV infection

Epithelial cells line the surface of the lungs, from the trachea, upper and lower respiratory tracts to the lung parenchyma where epithelial cell viability is vital for maintaining alveolar integrity. As IAV is an airborne respiratory virus, epithelial cells are readily exposed to IAV infection and can succumb to IAV-induced cell death through apoptosis^21^ or necrosis^44^. For example, IAV (H3N2) can induce apoptosis in the bronchiolar epithelial cell line NCI-H292 24 h post infection^64^. In mice, IAV infection simultaneously induces apoptosis and necrosis in the alveolar epithelial cell layer^54^. Therefore, a major consequence of IAV infection is the characteristic lung and tracheal epithelium damage evident in both human^65^ and murine^66^ infections. Epithelial cell death is thought to be a characteristic feature of lethal infections and the damage to the respiratory epithelium can directly lead to respiratory failure through lung oedema and impaired gas exchange^67^.

Besides direct infection, infiltrating monocyte-derived macrophages may also contribute to epithelial cell death and disease severity^68^. Exposure of macrophages to IAV can trigger secretion of TRAIL, which can induce apoptosis in surrounding epithelial cells via the death receptor 5^68,69^. This mechanism may explain why cell death of IAV-infected (NP positive) and uninfected (NP negative) alveolar epithelial cells can be observed during infection in vivo^54^. As bone marrow reconstitution with TRAIL deficient cells rescues epithelial cell viability and alveolar integrity during infection, IAV-driven macrophage-dependent epithelial cell apoptosis represents an additional mechanism that increases IAV disease lethality^68^. In contrast to macrophages, conventional natural killer (NK) cells may aid epithelial cell survival during IAV infection, as NK cells secrete IL-22 within the lung tissue to promote epithelial cell repair and regeneration^66^. However, this process may be dependent on NK cells evading infection as NK cells are also susceptible to IAV infection and IAV-induced apoptosis^70^.

During IAV infection, epithelial cells produce cytokines and chemokines including IL-6, IL-8 and CCL2 to facilitate both anti- and pro-viral responses^71,72^. Interestingly, cytokine production by epithelial cells may be regulated by IAV-induced apoptosis^64^. Although treatment of IAV (H3N2)-infected NCI-H292 epithelial cells with a pan-caspase inhibitor does not alter viral titres, impairment of apoptosis can significantly increase IL-6 and IL-8 production in vitro^64^. It should be noted that although pan-caspases inhibitors can impair apoptosis, mtDNA can still be released and promote proinflammatory cytokine production^73,74^. Thus, such assays using caspases inhibitors to assess cytokine production need to be evaluated with caution. Finally, in contrast to the previous study where pan-caspase inhibition did not alter viral titres^64^, viral replication has elsewhere been suggested as dependent on apoptosis. Inhibition of IAV (H1N1)-induced epithelial cell apoptosis using a pan-caspase inhibitor, caspase 3 depletion or by targeting the TRAIL pathway all significantly impaired viral replication and propagation, again highlighting the pro-viral role of apoptosis during IAV infection^36,75^. Taken together, IAV-infection of epithelial cells is predominantly detrimental for the host as it induces mainly apoptotic and necrotic cell death to facilitate viral propagation and comprises the integrity of the epithelial cell barrier.

### Macrophage and monocyte cell death during IAV infection

Alveolar macrophages (AMΦs) reside within the lung parenchyma where they are readily exposed to IAV during infection. In contrast to epithelial cells, AMΦs are poorly productively infected as they will rarely produce infectious virions^76,77^. Nevertheless, IAV infection is able to induce AMΦ cell death via apoptosis^20,78–80^. As AMΦs play key roles in viral clearance and immune regulation, depletion of AMΦs during IAV infection significantly compromises the host response and increases disease lethality^81^. Viral sensing by macrophages induces the release of a broad range of cytokines dictated by the IAV strain^71,82^. Specifically, macrophages are the main source of IFN-β production during IAV pathogenesis although, this is dependent on macrophage evasion of IAV-induced apoptosis^72,83,84^. To promote survival and facilitate cytokine release, the AMΦ pattern-recognition receptor NLRX1 may interact with the pro-apoptotic IAV protein PB1-F2 to impair apoptosis, in turn promoting the production of IFN-β^84^. Interestingly, IFN-β secretion by AMΦs may be a key component of IAV-macrophage-dependent epithelial cell death pathway (as described above), as exposure to IAV and secretion of IFN-β by AMΦs can induce AMΦ TRAIL expression through autocrine signalling and induce epithelial cell death^69^. It is possible that viral-sensing by AMΦs may induce epithelial cell apoptosis as a host defence mechanism to pre-emptively reduce viable hosts susceptible to IAV infection.

IAV infection and apoptosis in human and murine AMΦs also induces TNF-α secretion, which may indirectly promote monocyte migration^80,85^. Although IAV infection of AMΦs is comparable for mammalian and avian IAV strains, avian strains are associated with enhanced apoptosis^20^. Furthermore, in contrast to epithelial cell IL-6 and AMΦ IFN-β production where expression was limited by apoptosis^64,84^, avian IAV-induced AMΦ apoptosis was associated with increased TNF-α production^20^. Similarly, chemokine secretion by IAV infected monocytes may also be apoptosis-dependent. Monocytes play both beneficial and detrimental roles during IAV infections by facilitating CD8 T cell priming and enhancing immunopathology, respectively^86,87^. IAV infection of monocytes can induce apoptosis and the production of inflammatory cytokines and chemokines such as IL-1, IL-6, TNF-α and CXCL10, with the latter significantly impaired by the addition of a pan-caspase inhibitor^72,79,88^. Although the mechanistic link between caspase activation and cytokine/chemokine release remains undefined, these findings further highlight the importance of apoptosis in regulating virus-induced immune responses^79^.

### Neutrophil cell death during IAV infection

During IAV infection, neutrophils infiltrate into the lung approximately 2 days post infection and play key roles in facilitating viral clearance and disease resolution^81,89,90^. Although there are limited studies directly characterising neutrophil cell death and its consequence during IAV infection, IAV-induced apoptosis has been documented in human neutrophils in vitro^91^, and in murine^31^ and canine infections^92^ in vivo. Interestingly, IL-6 may promote neutrophil survival during IAV (H1N1)-infections as the addition of IL-6 to in vitro infection assays is sufficient to maintain neutrophil viability, and IL-6 deficiency significantly enhances viral titres, reduces neutrophil levels and overall heightens disease severity^31^. Although IAV can downregulate the anti-apoptotic factors Mcl-1 and Bcl-X_L_ to induce neutrophil apoptosis, IL-6 is able to restore Mcl-1 and Bcl-X_L_ levels to limit apoptosis and maintain neutrophil survival^31^. Together, these findings demonstrate a key role for IL-6 during infection and further supports the anti-viral role of neutrophils in aiding IAV clearance and disease resolution^31,93,94^. Neutrophils have also been suggested as a major driver of IAV-induced lethality where depletion of neutrophils could increase mouse survival rate during H1N1 IAV infection^90,95^. Although neutrophils are required for the early clearance of IAV, heightened numbers of recruited neutrophils may lead to the accumulation of inflammatory/tissue damaging signalling networks^90^. Therefore, in such settings the initiation of neutrophil apoptosis and thus reduction of neutrophil levels may be advantageous to limit inflammation and bystander tissue injury.

## The aftermath of cell death: consequences of cell debris formation

**Fig. 3:**
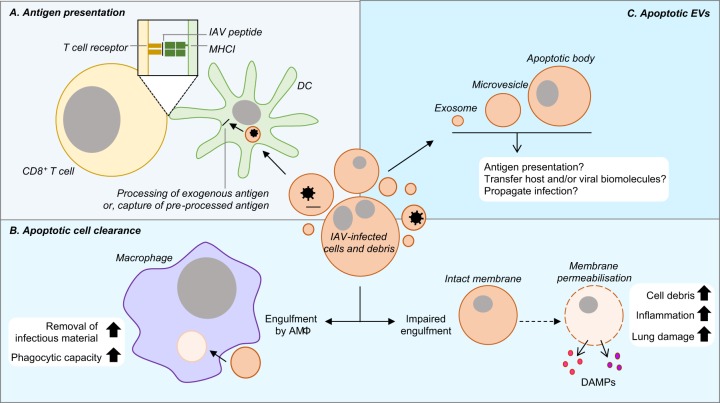
Functions and consequences of cell debris formation during IAV infection. **a** Dead cells and debris generated from IAV-infected cells may be taken up by DCs, where engulfed viral proteins may be cross-presented to IAV-specific CD8^+^ T cells. **b** Macrophages can efficiently phagocytose cell debris to remove infectious material. However, if engulfment is impaired apoptotic particles can lyse through secondary necrosis, releasing an abundance of DAMPs that can exacerbate inflammation and lung damage. **c** Whether apoptotic EVs also contribute to viral pathogenesis is unknown. However, it is possible that such EVs could transfer viral molecules and contribute to antigen presentation or viral propagation

As IAV induces a significant degree of cell death within the respiratory tract and lung parenchyma, the presence of both dying cells and their debris may significantly impact disease resolution (Fig. 3). Therefore, it is important to highlight that the consequences of infection do not end at the induction of cell death.

### IAV-induced cell death and antigen presentation

The generation of an antigen-specific adaptive immune response is essential for the clearance of viral infections. Thus, the ability of apoptotic or necrotic pathogen-infected cells to promote antigen presentation is advantageous for the host. Initially, it was demonstrated that immature CD83^−^ DCs, a professional antigen presenting cell (APC) type, could phagocytose and acquire antigens from IAV-infected, apoptotic monocytes in vitro^96^. Phagocytosis of infected-cells occurs approximately 2–4 h post apoptosis and induces the maturation of DCs, allowing the presentation of apoptotic cell-derived IAV antigen to IAV-specific CD8^+^ T cells^96,97^. As the addition of a pan-caspase inhibitor to in vitro assays impaired the generation of CD8^+^ cytotoxic T cells, this process was suggested to be dependent on the induction of apoptosis itself, likely through the exposure of ‘eat-me’ signals on IAV-infected apoptotic cells to promote phagocytosis^97^. In line with this, the lipid mediator prostaglandin E_2_ (PGE2) was shown to impair the induction of IAV-induced MΦ apoptosis^98^. As a consequence, PGE2^−/−^ mice infected with IAV exhibited an increase in apoptosis and enhanced T cell-mediated immunity, further highlighting the role of apoptotic cells in aiding antigen presentation and anti-viral immunity^98^.

In contrast to apoptosis, primary necrotic cells were shown to be superior at inducing a T cell response^99^. Although immature DCs could phagocytose both apoptotic and necrotic cells equally, necrotic cell uptake resulted in a heightened capacity to stimulate T cells, possibly through a stimulatory factor released by necrotic cells^99^. Finally, it is interesting to note that previously processed antigen may be acquired by DCs through apoptotic cell uptake. By utilising IAV-infected, MHC match or mismatched apoptotic cells which were either TAP competent or deficient (either able or unable to present endogenous peptides), and either TAP competent or deficient DCs as antigen cross-presenting APCs, two possible pathways have been suggested whereby (i) DCs can acquire IAV antigen from the apoptotic cell for peptide processing and presentation; and (ii) DCs can acquire IAV antigen previously processed within the ER of the apoptotic cell for MCHI loading^100^. Overall, DC cross-presentation may be important during influenza infection as antigens from phagocytosed cells and debris can contribute to the induction of a robust T cell response.

### IAV-induced cell death and apoptotic cell clearance

If apoptotic cells are not rapidly removed, the plasma membrane can rupture and release a series of inflammatory molecules through secondary necrosis (Fig. 3)^101^. Therefore, the efficient clearance of IAV-infected apoptotic debris by phagocytes is essential to avoid further exacerbation of inflammation. After the initiation of cell death on day 2 post infection, phagocytosis of apoptotic IAV-infected cells by macrophages can be detected in the BAL fluid and lung tissue^93,102^. Moreover, uptake of infected cells by AMΦs can be impaired by the addition of annexin A5, a protein which binds to the ‘eat-me’ signal PtdSer, indicating macrophages are able to undergo PtdSer-mediated phagocytosis during IAV-infection^102–104^. Interestingly, prior exposure of macrophages to IAV may increase their ability to phagocytose apoptotic cells^79,93^. It is suggested that apoptotic IAV-infected cells could release stimulatory factor(s) to enhance the phagocytosis efficiency of surrounding macrophages, as supernatant of IAV-infected but not UV-irradiated apoptotic cells could enhance the phagocytic capacity of AMΦs^93^. Together, these results suggest a possible host defence mechanism to ensure that infectious or harmful cell debris are rapidly removed during infection. Notably, impairment of phagocytosis during IAV infection by the administration of annexin A5 could significantly enhance disease pathology in mice and decrease survival rate^102^. Similarly, the lack of AMΦs in GM-CSF^−/−^ mice can impair apoptotic cell clearance during IAV infection, resulting in an accumulation of dead cells/debris which may contribute to lung damage^105^. It is suggested that the clearance of apoptotic debris by AMΦs may be mediated by TLR4, as lack of TLR4 could impair phagocytosis and enhance disease pathogenesis^93^. However, these findings were not reproduced in in vitro assays^93^. Nevertheless, the uptake of dying cells during infection is likely to aid the removal of infectious material and the resolution of inflammation.

### IAV-induced cell death and apoptotic cell-derived extracellular vesicles?

Finally, a concept yet to be defined is the role of apoptotic cell-derived extracellular vesicles (EVs) during IAV infection (Fig. 3). During the final stages of apoptosis, the apoptotic cell can fragment to generate membrane-bound vesicles known as apoptotic bodies^106^. Apoptotic cells may also generate smaller EVs including exosomes^107^ and microvesicles^108^. All EVs can harbour a series of biomolecules including DNA, RNA and protein to mediate intercellular communication^5,109,110^. Therefore, whether such vesicles may harbour viral materials and infectious virions and contribute to the progression of IAV infection would be of interest to determine.

## Conclusions

As IAV can hijack a series of host cellular processes such as cell death machinery, the development of novel therapeutics targeting cell death during IAV infection has previously been suggested^111^. However, here we outlined and discussed the complexity of IAV-induced cell death. IAV can promote apoptosis, necrosis, necroptosis and pyroptosis, and the molecular mechanisms driving these pathways are varied. Furthermore, the mechanism of cell death may also be cell type-specific and/or dependent on a specific IAV strain. Therefore, current research highlights the difficulties in developing anti-viral therapeutics targeting cell death processes as such approaches may only target a small proportion of all virus-infected cells. As apoptosis is generally considered as an anti-inflammatory process, therapeutics designed to skew cell death from pro-inflammatory pathways such as necroptosis to apoptosis has been postulated to be beneficial. However, this scenario is much more complex as apoptosis may actually enhance viral replication and dissemination whilst limiting the pro-inflammatory immune response. Overall, an in-depth and context-dependent understanding of cell death pathways during the progression of IAV infection may allow us to better identify therapeutically beneficial targets that do not inadvertently increase disease pathogenicity through other mechanisms.
